# Effects of Person-Centered Care Using a Digital Platform and Structured Telephone Support for People With Chronic Obstructive Pulmonary Disease and Chronic Heart Failure: Randomized Controlled Trial

**DOI:** 10.2196/26794

**Published:** 2021-12-13

**Authors:** Lilas Ali, Sara Wallström, Andreas Fors, Emmelie Barenfeld, Eva Fredholm, Michael Fu, Mahboubeh Goudarzi, Hanna Gyllensten, Irma Lindström Kjellberg, Karl Swedberg, Lowie E G W Vanfleteren, Inger Ekman

**Affiliations:** 1 Sahlgrenska Academy Institute of Health and Care Sciences University of Gothenburg Gothenburg Sweden; 2 Centre for Person-Centred Care University of Gothenburg Gothenburg Sweden; 3 Psychiatric Department Sahlgrenska University Hospital Gothenburg Sweden; 4 Region Västra Götaland Research and Development Primary Health Care Gothenburg Sweden; 5 Department of Occupational Therapy and Physiotherapy Sahlgrenska University Hospital Gothenburg Sweden; 6 The Swedish Heart & Lung Foundation Stockholm Sweden; 7 Department of Molecular and Clinical Medicine Sahlgrenska Academy University of Gothenburg Gothenburg Sweden; 8 Chronic Obstructive Pulmonary Disease Center Department of Respiratory Medicine and Allergology Sahlgrenska University Hospital Gothenburg Sweden; 9 Department of Internal Medicine and Clinical Nutrition Institute of Medicine, Sahlgrenska Academy University of Gothenburg Gothenburg Sweden

**Keywords:** chronic heart failure, chronic obstructive pulmonary disease, digital platform, eHealth, patient-centered care, person-centered care, randomized controlled trial, telehealth

## Abstract

**Background:**

Chronic obstructive pulmonary disease (COPD) and chronic heart failure (CHF) are characterized by severe symptom burden and common acute worsening episodes that often require hospitalization and affect prognosis. Although many studies have shown that person-centered care (PCC) increases self-efficacy in patients with chronic conditions, studies on patients with COPD and CHF treated in primary care and the effects of PCC on the risk of hospitalization in these patients are scarce.

**Objective:**

The aim of this study is to evaluate the effects of PCC through a combined digital platform and telephone support for people with COPD and CHF.

**Methods:**

A multicenter randomized trial was conducted from 2018 to 2020. A total of 222 patients were recruited from 9 primary care centers. Patients diagnosed with COPD, CHF, or both and with internet access were eligible. Participants were randomized into either usual care (112/222, 50.5%) or PCC combined with usual care (110/222, 49.5%). The intervention’s main component was a personal health plan cocreated by the participants and assigned health care professionals. The health care professionals called the participants in the intervention group and encouraged narration to establish a partnership using PCC communication skills. A digital platform was used as a communication tool. The primary end point, divided into 2 categories (improved and deteriorated or unchanged), was a composite score of change in general self-efficacy and hospitalization or death 6 months after randomization. Data from the intention-to-treat group at 3- and 6-month follow-ups were analyzed. In addition, a per-protocol analysis was conducted on the participants who used the intervention.

**Results:**

No significant differences were found in composite scores between the groups at the 3- and 6-month follow-ups. However, the per-protocol analysis of the 3-month follow-up revealed a significant difference in composite scores between the study groups (*P*=.047), although it was not maintained until the end of the 6-month follow-up (*P*=.24). This effect was driven by a change in general self-efficacy from baseline.

**Conclusions:**

PCC using a combined digital platform and structured telephone support seems to be an option to increase the short-term self-efficacy of people with COPD and CHF. This study adds to the knowledge of conceptual innovations in primary care to support patients with COPD and CHF.

**Trial Registration:**

ClinicalTrials.gov NCT03183817; http://clinicaltrials.gov/ct2/show/NCT03183817

## Introduction

### Background

Chronic obstructive pulmonary disease (COPD) and chronic heart failure (CHF) are known for their high mortality and severe impact on daily living activities [1-3]. Although pharmacological therapy has dramatically improved outcomes over the past decade, patients still perceive a high symptom burden and acute worsening of events. Therefore, self-management strategies that enhance self-efficacy are crucial to optimize [4] and strengthen preventive approaches in primary care [5]. Digital solutions have been suggested as a safe option for addressing health care challenges and promoting self-management of chronic conditions such as COPD and CHF [6-8]. However, most digital solutions lack user involvement in the development of the platform [9].

Person-centered care (PCC) is an approach based on ethical principles by which a contractual agreement is formed involving the patient as an active partner in the care and decision-making process [10]. To support the operationalization of person-centered ethics in clinical practice, a framework was developed by the Gothenburg Centre for Person-Centred Care. This Gothenburg Centre for Person-Centered Care framework underlines the importance of cocreated care between patients and health care professionals (HCPs; eg, registered nurses and a physiotherapist) based on the patient’s narrative, which identifies personal resources and potential barriers together with medical status [10,11]. A central concept of PCC is self-efficacy, that is, a person’s conviction in his or her ability to manage challenges and complete a task successfully [12]. Enhanced self-efficacy has been shown to improve disease management and clinical outcomes, including health status in patients with chronic diseases [13], physical functioning in patients with COPD and CHF [14], and daily living in patients with COPD [15]. Thus, HCPs need to target patients’ self-efficacy beliefs to perform desired activities and support them in taking responsibility for engaging in their care [16]. Previous research has shown that PCC increases the self-efficacy of patients [17-19]. PCC via telephone is also thought to mitigate worsening self-efficacy in COPD and CHF, indicating that a partnership could be established between patients and HCPs without face-to-face contact [20].

Several studies have used telemedicine and digital interventions for people with COPD and CHF [21,22]; however, PCC was not part of their design, and the results were mixed [23].

### Objective

We hypothesize that PCC principles that include a digital platform and structured telephone support for people with COPD and CHF would reduce the need for primary care and hospital admission and improve self-efficacy through collaboration in the care process. Therefore, the aim of this study is to evaluate the effects of PCC through a combined digital platform and telephone support for people with COPD and CHF.

## Methods

### Design, Participants, and Setting

**Figure 1:**
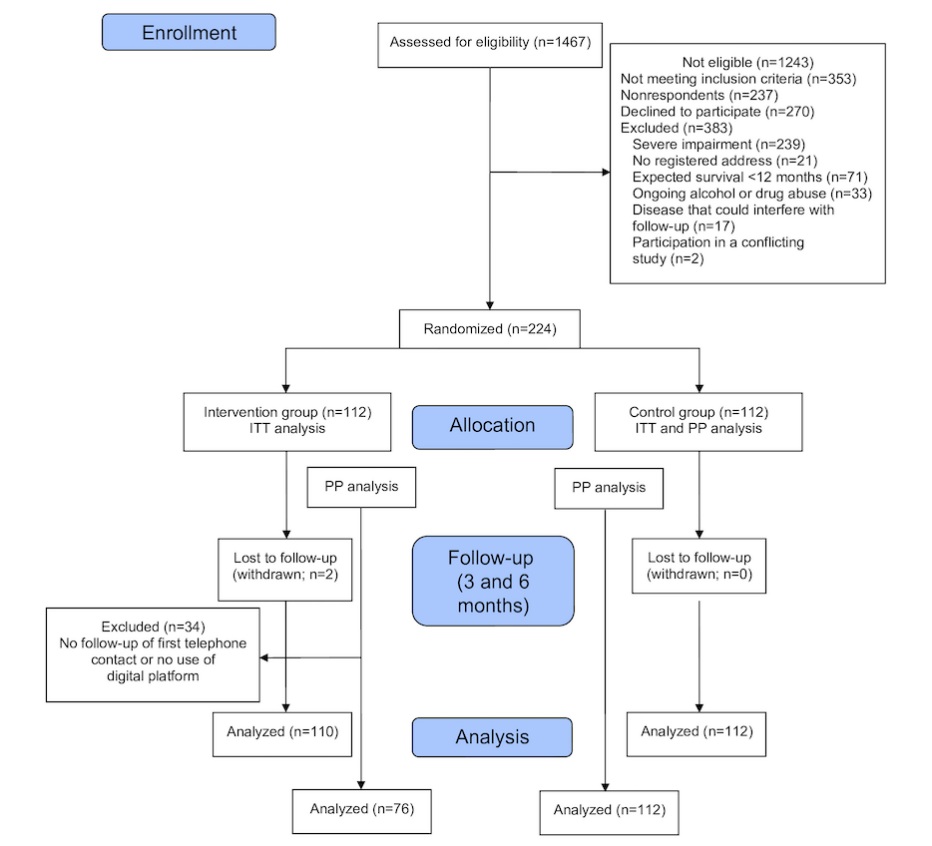
CONSORT flowchart. ITT: intention-to-treat; PP: per-protocol.

Study participants were recruited from 9 primary health care centers in Gothenburg from August 2017 to June 2019. The study, including consent forms for participants, was approved by the regional ethics board in Gothenburg (Dnr 063-17 and T613-18). The inclusion criteria were a diagnosis of COPD (J43.0, J44.0-J44.9) or CHF (I50.0-I50.9), being listed at one of the participating primary health care centers, understanding written and spoken Swedish, and having access to a device with an internet connection. The exclusion criteria were severe impairment (cognitive or other) that prevented the individual from using eHealth support, no registered address (follow-up questionnaires were sent by mail), expected survival of <12 months, ongoing documented diagnosis of alcohol or drug abuse, diseases that could interfere with follow-up (ie, multimorbidity), and participation in a conflicting study. A flowchart of the study participants is given in Figure 1.

### Enrollment and Randomization

Designated HCPs screened medical records at the 9 participating centers for potential patients diagnosed with COPD, CHF, or both. Eligible participants were sent an information letter about the study with an invitation to contact the HCPs for more information. If the participants did not contact an HCP within 2 weeks after the information letter had been sent out, they were contacted by phone for further details and asked to participate. Patients who accepted the invitation to participate were sent a consent form together with a prepaid return envelope by mail. Upon receipt of the signed consent form, patients were randomized into either standard care or PCC in addition to standard care. Randomization was based on a computer-generated list created by a third party and stratified by age (<65 or ≥65 years) and diagnostic group (COPD, CHF, or COPD and CHF). All participants were informed of their allocation by phone, a call not included as part of the intervention.

### Usual Care

Usual care was managed as per the physician’s judgment based on current guidelines, for example, medicine adjustments [24,25]. The usual care group had no follow-up phone conversations.

### Intervention

The intervention, comprising PCC using a combined digital platform and structured telephone support system, was provided in addition to usual care for 6 months. The structured telephone support program comprised an optional number of phone calls that included a health plan cocreated and followed up by patients and HCPs, which is consistent with person-centered principles. The digital platform was aimed at supporting communication between phone calls and providing access to shared documentation (health plans and self-ratings) and reliable information sources. The digital platform was developed using a participatory design with user involvement. The method draws on the user’s *tacit knowledge,* that is, their implicit or unarticulated knowledge learned and transmitted through experience and apprenticeship, for example, by taking part in this project. The researchers had workshops together with the HCPs, patient partners, and experts in which the platform and intervention were discussed and developed [26].

HCPs assisted participants in the intervention group in creating a log-in to the digital platform that they could access during the study period and described its features. In the first telephone conversations, the HCPs encouraged narration. They established a partnership using communication skills such as listening to the participants’ narratives about daily life events and how they were affected by their condition. The next step entailed cocreating a health plan based on patient narratives through discussion and agreement, including patient goals, resources, and needs. Usually, the health plan contained information about what the participants had talked about, how they felt, what goals they had, and what they wanted to accomplish. The health plan also included information on the participants’ capabilities and resources that could be used to help them reach their respective health goals. The health plan was written jointly at the initiative of the HCPs or the patients and uploaded to the digital platform by either the HCPs or the patients.

The HCPs and patients jointly scheduled the date of the follow-up meetings based on the preferences of the patients. The health plan served as a point of departure for the impending conversations and communication via the platform. The participants and HCPs had access to the platform during the 6-month study period. The health plan was considered and revised during each follow-up phone call and when needed (eg, if the participants spontaneously contacted the HCPs). The fidelity of the intervention was ensured by meetings and seminars on a regular basis, constructive discussions and education on PCC, and person-centered communication and monitoring of the HCPs by researchers and experts in their respective fields. The review group comprised senior and junior researchers and patient representatives. In addition, the HCPs reviewed some of each other’s telephone calls and health plans. A total of 5 HCPs were involved in the intervention: 3 (60%) registered nurses, 1 (20%) occupational therapist, and 1 (20%) physiotherapist. Years of work experience ranged from 6 to 26 years for the HCPs.

The platform contained functionalities for 2-way communication through private messages; the possibility to rate daily symptoms, such as shortness of breath and tiredness, to be visualized as trend graphs; and an archive of the health plans. The participants could invite and give customized access to the platform to any person they wanted, such as informal carers, family, or friends. They could also access links to relevant websites containing information on COPD and CHF provided by patient organizations (eg, the Heart and Lung Association) and the Swedish national support guide to an online peer-to-peer support group. A detailed description of the intervention has been published elsewhere [27].

### Collection of Data and Outcome Measures

Data were collected through questionnaires and from medical records at inclusion and 3 and 6 months later. Questionnaires were mailed to all participants together with a prepaid return envelope. If the questionnaires were not returned within 2 weeks, reminders were given by phone. New questionnaires and return envelopes were sent out if needed. Approximately 6.8% (15/222) of participants (11/110, 10% in the intervention group and 4/112, 3.6% in the control group) did not return their questionnaires at the 3-month follow-up. At the 6-month follow-up, 8.6% (19/222) of participants (13/110, 11.8% in the intervention group and 6/112, 5.4% in the control group) did not return their questionnaires despite being reminded.

The primary end point was a composite score of general self-efficacy (GSE) changes and hospitalization or death 6 months after randomization into each group.

A patient was classified as improved if GSE increased by ≥5 units, the patient was not hospitalized because of COPD or CHF, and the patient did not die. A patient was classified as deteriorated if GSE decreased by ≥5 units, the patient was admitted for unscheduled reasons because of COPD or CHF, or the patient died because of any cause.

Those who neither improved nor deteriorated were considered unchanged. GSE was assessed using the GSE scale, a 10-item scale designed to measure a sense of personal competence in dealing with stressful situations (eg, handling unforeseen situations and finding possible solutions to problems). The GSE scale has been widely tested and used internationally; the Swedish version has shown high internal consistency (Cronbach α=.90) [28]. The 10 items are rated on a 4-point scale, with total scores ranging from 10 to 40 [29]. An increase of 5 units has been suggested as a threshold for minimal clinically meaningful change [18,20].

### Statistical Methods

A sample size of at least 91 participants in each group was needed to achieve a power of 80% based on a *P* value of .05 (2-tailed) to detect an increase in the composite score from 20% in the control group to 40% in the intervention group.

Descriptive and comparative statistics were used to characterize the study groups. Group differences were calculated using the Pearson chi-square test for categorical variables, the Fisher exact test for dichotomous variables, and the independent 2-tailed Student *t* test for continuous variables. Between-group differences in the composite score were tested using the Fisher exact test for the dichotomous version and the Mantel-Haenszel chi-square test for the ordered categorical version. Binary logistic regression was used to calculate odds ratios with 95% CIs for the dichotomous version of the composite score. The Student *t* test was used to compare the mean change in GSE scores between groups. Between-group differences in improvement of ≥5 points on the GSE scale were calculated in the same way as the dichotomous version of the composite score. Bivariate correlations were computed using Pearson *r*. Missing outcome data for the 3- and 6-month follow-ups were imputed using the last value carried forward. Sensitivity analyses were conducted to assess robustness. Both intention-to-treat (ITT) and per-protocol (PP) analyses were conducted. The PP group included participants with at least one PCC phone call and at least one health plan who logged into the platform and used at least one of its functions. The significance level was set at *P*<.05 (2-sided).

## Results

### Overview

In total, 224 participants were randomly assigned to either the control or the intervention group, of which 2 participants withdrew consent, leaving 222 participants (112/222, 50.5% in the control group and 110/222, 49.5% in the intervention group). The baseline characteristics are presented in Table 1. The study population was 53.6% (119/222) men and 46.4% (103/222) women, with a mean age of 70.8 (SD 9.4) years. Approximately 51.8% (115/222) participants had COPD, 38.3% (85/222) had CHF, and 9.9% (22/222) had both. Of the 222 participants, 32 (14.4%) were current smokers. The treatment and control groups were similar at baseline, except that significantly more participants in the control group were married or living with a partner than in the intervention group (*P*=.01). However, this difference did not remain when comparing the control and PP groups (*P*=.07). There were no significant differences between the groups in medical histories (eg, diagnosis, previous cardiovascular disease, or stages of COPD; Table 1).

**Table 1:** Baseline participant characteristics (N=222).

Characteristics			Control group (n=112)		ITT^a^ group (n=110)				PP^b^ group (n=76)		
					Value		*P* value		Value		*P* value
Age (years), mean (SD)			70.4 (9.1)		71.1 (9.8)		.59		70.1 (9.1)		.84
Women, n (%)			52 (46.4)		51 (46.4)		.99		31 (40.8)		.46
BMI, mean (SD)			28.2 (5.1)^c^		29.0 (5.4)^d^		.33		29.0 (5.3)^e^		.35
GSE^f^ score, mean (SD)			31.1 (6.2)		31.0 (5.4)^g^		.99		30.9 (5.4)^h^		.83
**Civil status, n (%)**											
	Living alone	25 (22.3)		42 (38.2)		.01		25 (34.2)		.07	
	Married or living with partner	87 (77.7)		68 (61.8)		.01		49 (64.5)		.07	
**Diagnosis, n (%)**											
	CHF^i^	43 (38.4)		42 (38.2)		.88		27 (35.5)		.49	
	COPD^j^	59 (52.7)		56 (50.9)		.88		38 (50)		.49	
	CHD and COPD	10 (8.9)		12 (10.9)		.88		11 (14.5)		.49	
**Stage of COPD, n (%)**											
	Stage 1	16 (27.6)		16 (26.2)		.99		10 (22.2)		.94	
	Stage 2	36 (62.1)		38 (62.3)		.99		30 (66.7)		.94	
	Stage 3	5 (8.6)		6 (9.8)		.99		4 (8.9)		.94	
	Stage 4	1 (1.7)		1 (1.6)		.99		1 (2.2)		.94	
**Education level, n (%)**											
	Compulsory	26 (23.2)		38 (34.5)		.13		26 (34.2)		.23	
	Secondary school	32 (28.6)		25 (22.7)		.13		18 (23.7)		.23	
	Vocational college	21 (18.8)		25 (22.7)		.13		17 (22.4)		.23	
	University	33 (29.5)		22 (20)		.13		15 (19.7)		.23	
**Smoking, n (%)**											
	Current smoker	19 (17)		13 (11.8)		.51		8 (10.5)		.47	
	Previous smoker	63 (56.3)		63 (57.3)		.51		46 (60.5)		.47	
	Never smoked	30 (26.8)		34 (30.9)		.51		22 (28.9)		.47	
**Medical history, n (%)**											
	Previous MI^k^	12 (10.7)		15 (13.6)		.54		10 (13.2)		.65	
	Previous angina	8 (7.1)		9 (8.2)		.81		8 (10.5)		.44	
	Atrial fibrillation	31 (27.7)		39 (35.5)		.25		26 (34.2)		.42	
	Hypertension	68 (60.7)		76 (69.1)		.21		49 (64.5)		.65	
	CABG^l^	3 (2.7)		2 (1.8)		.99		2 (2.6)		.99	
	Previous stroke	6 (5.4)		11 (10)		.22		10 (13.2)		.07	
	Diabetes	19 (17)		23 (20.9)		.50		18 (23.7)		.27	
	CRT^m^	1 (0.9)		1 (0.9)		.99		1 (1.3)		.99	
	Pacemaker	7 (6.3)		6 (5.5)		.99		4 (5.3)		.99	
Previous spirometry <6 months before inclusion, n (%)			19 (31.7)		14 (22.6)		.31		11 (25)		.52
<6 months FEV^n^ 1% of expected value, mean (SD)			68.8 (17.3)^o^		67.6 (16.9)^p^		.85		64.1 (16.0)^q^		.47
Previous spirometry 6-12 months before inclusion, n (%)			8 (13.3)		13 (21)		.34		11 (25)		.20
6-12 months FEV 1% of expected value, mean (SD)			66.8 (14.0)^r^		70.5 (15.6)^s^		.61		68.7 (16.4)^t^		.81
Previous spirometry >12 months before inclusion, n (%)			31 (54.4)		33 (55)		.99		22 (52.4)		.99
>12 months FEV 1% of expected value, mean (SD)			71.0 (16.4)^u^		66.1 (16.2)^v^		.24		64.1 (16.2)^w^		.14

^a^ITT: intention-to-treat.

^b^PP: per-protocol.

^c^n=91.

^d^n=87.

^e^n=60.

^f^GSE: general self-efficacy.

^g^n=107.

^h^n=73.

^i^CHF: chronic heart failure.

^j^COPD: chronic obstructive pulmonary disease.

^k^MI: myocardial infarction.

^l^CABG: coronary artery bypass graft.

^m^CRT: cardiac resynchronization therapy.

^n^FEV: forced expiratory volume.

^o^n=19.

^p^n=13.

^q^n=11.

^r^n=7.

^s^n=12.

^t^n=10.

^u^n=30.

^v^n=33.

^w^n=21.

During the 6-month intervention, the ITT group had a median of 4 (range 0-11) telephone conversations with the HCPs. Of those, a median of 3 (range 0-7) was within 90 days of randomization. The PP group had a median of 4 (range 1-11) telephone conversations during the study period, with a median of 3 (range 1-7) within 90 days of randomization. No statistically significant correlations were detected between the number of calls and changes in GSE.

### Effects

No significant differences in composite scores (improved vs deteriorated or unchanged) at the 3- or 6-month follow-ups were observed between the groups. However, a significant difference in the PP analysis was noted at the 3-month follow-up (*P*=.047). The analysis confirmed this result using the composite score (improved, unchanged, or deteriorated; *P*=.04). However, none of these differences could be sustained at the 6-month follow-up (*P*=.24 and *P*=.50; Table 2). We found no differences in composite scores between patients with COPD, CHF, or COPD and CHF. During the 6-month intervention, there were 4 recorded hospitalizations: 3 (75%) in the intervention group and 1 (25%) in the control group. There were no participant deaths during the intervention.

**Table 2:** Composite scores at the 3- and 6-month follow-up.

Time and composite score				Control (n=112)		Intention-to-treat (n=110)^a^							Per-protocol (n=76)^a^				
						Value		OR^b^ (95% CI)		*P* value		Value			OR (95% CI)		*P* value
**3 months**																	
	**Composite score 1^c^, n (%)**																
		Improved	10 (8.9)		15 (14)		1.663 (0.712-3.884)		.24		14 (19.2)			2.420 (1.011-5.792)		.047^d^	
		Deteriorated or unchanged	102 (91.1)		92 (86)		1.663 (0.712-3.884)		.24		59 (80.8)			2.420 (1.011-5.792)		.047^d^	
	**Composite score 2^e^, n (%)**							—^f^		—					—		
		Improved	10 (8.9)		15 (14)						14 (19.2)					.04^d^	
		Unchanged	89 (79.5)		83 (77.6)						54 (74)					.04^d^	
		Deteriorated	13 (11.6)		9 (8.4)						5 (6.8)					.04^d^	
**6 months**																	
	**Composite score 1^c^, n (%)**																
		Improved	13 (11.6)		16 (15)		1.339 (0.611-2.936)		.47		13 (17.8)			1.650 (0.717-3.795)		.24	
		Deteriorated or unchanged	99 (88.4)		91 (85)		1.339 (0.611-2.936)		.47		60 (82.2)			1.650 (0.717-3.795)		.24	
	**Composite score 2^e^, n (%)**							—		—					—		
		Improved	13 (11.6)		16 (15)						13 (17.8)					.50	
		Unchanged	83 (74.1)		74 (69.2)						49 (67.1)					.50	
		Deteriorated	16 (14.3)		17 (15.9)						11 (15.1)					.50	

^a^3 missing values (no general self-efficacy score at baseline).

^b^OR: odds ratio.

^c^Composite score dichotomized into improved vs deteriorated or unchanged.

^d^Significant at *P*<.05.

^e^Composite score improved vs unchanged vs deteriorated.

^f^The statistical test used (Mantel-Haenszel chi-square test) does not generate odds ratio or 95% CI.

Differences between the groups at 3 months were driven mainly by changes in the GSE scale. The PP group had a mean improvement of 0.941 (SD 4.4) points, whereas the control group had a mean reduction of 0.568 points (SD 4.6; *P*=.03). At the 3-month follow-up, 19.2% (14/76) of the participants in the PP group versus 8.9% (10/112) of those in the control group had an improvement of ≥5 points on the GSE scale, indicating that the PP group was more than twice as likely to have a clinically meaningful improvement in GSE compared with the control group (Table 3).

**Table 3:** Change in general self-efficacy (GSE) from baseline and at the 3- and 6-month follow-ups.

Follow-up			Control (n=112)		ITT^a^ (n=110)^b^						PP^c^ (n=76)^d^				
					Value		OR^e^ (95% CI)		*P* value		Value		OR (95% CI)		*P* value
**3 months**															
	Change in GSE score, mean (SD)	−0.568 (4.6)		0.544 (4.0)		2.261 to 0.038^d^		.06		0.941 (4.4)		−2.842 to −0.174^d^		.03^f^	
	Improvement ≥5 points, n (%)	10 (8.9)		15 (14)		1.663 (0.712 to 3.884)		.24		14 (19.2)		2.420 (1.011 to 5.792)		.047^f^	
**6 months**															
	Change in GSE score, mean (SD)	−0.397 (4.5)		0.127 (4.3)		−1.689 to 0.642^d^		.38		0.384 (4.4)		−2.103 to 0.542^d^		.25	
	Improvement ≥5 points, n (%)	13 (11.6)		16 (15)		1.339 (0.611 to 2.936)		.47		13 (17.8)		1.650 (0.717 to 3.795)		.24	

^a^ITT: intention-to-treat.

^b^3 missing values (no general self-efficacy score at baseline).

^c^PP: per-protocol.

^d^Only 95% CI shown as the statistical test (Student *t* test) does not generate odds ratio.

^e^OR: odds ratio.

^f^Significant at *P*<.05.

## Discussion

### Principal Findings

This study shows that a 6-month intervention with PCC using a combined digital platform and structured telephone support system does not improve outcome as assessed by a composite end point of change on the GSE score and absence of hospitalization or death. However, the PP analysis, which only included those who used the intervention, showed a difference in composite scores at 3 months. However, this difference did not hold at the 6-month follow-up. The difference at 3 months was primarily because of an improvement in the GSE scale score. PP analyses are usually data-driven and thus, of lower evidence than prespecified analyses but are important to explore the mechanisms behind the effects of treatment and care interventions [30].

Several explanations have been proposed to account for why the ITT analysis showed no significant differences and why the PP analysis only showed differences at the 3-month follow-up. The participants had a relatively stable disease, as illustrated by the few disease-related hospitalizations and the high self-reported GSE at baseline (mean score 31.1; Table 1). In contrast, population studies have reported a mean GSE score of 29 [29]. A previous study from our group targeting patients with more severe forms of COPD and CHF demonstrated more pronounced positive results from a similar intervention. In that study, the participants had lower GSE (mean score of 28) at baseline and therefore, had more margin for improvement [20]. Previous studies have highlighted the difficulties in showing the significance of an intervention when the participants’ score on outcome measurements at baseline (ie, ceiling effects) was high [31].

The intervention’s timing might have played a role in the lack of significant differences between the groups. The intervention sought to reach out with a preventive tool to support self-management and, therefore, recruited participants from primary care centers. Thus, unlike our previous interventions that mainly recruited participants during an unplanned hospitalization, in this intervention, patients were targeted in their everyday lives. In those interventions, hospitalization was an exact starting point, and many were eager to recover their health after a period of deterioration [18,20]. That the intervention’s timing is important for its meaningfulness was also confirmed by the findings from the participant interviews [32]. Moreover, previous research has pointed out the importance of including participants in need of an intervention to achieve favorable outcomes [31]. In this study, screening was not performed to determine which participants might be most suitable for the intervention.

The intervention showed an effect after 3 months in the PP analysis but not after 6 months. This result is likely because of the participants’ initial high degree of communication with the HCPs and the fact that the increase in GSE caused by the intervention attenuated over time. At least two phone meetings were initially made, one occurring when the patients were contacted and asked to participate and the other during the health plan’s joint formulation. As the median number of calls was 4, many of the participants had few conversations with HCPs after the first 2 calls. The intervention did not include mandatory follow-up or booster calls. If this had been done, the effect of the intervention might have been maintained. Nevertheless, no statistically significant associations were found between the number of calls and changes in the GSE. Associations might have been present; however, the study was underpowered to detect them. There may also be other explanations for the lack of effect after 6 months, such as lack of motivation and worsening of COPD and CHF; however, these variables were not collected.

Another possibility for the ITT analysis not detecting any differences is that the participants did not use the digital platform to its fullest extent. A reason for this lack of difference may be that the participants did not feel the need to use the digital platform when feeling stable [32]. Even if used, the digital platform might not have added enough support to improve GSE. These possibilities are important to explore further to find the best technique for future digital communication between HCPs and participants. It can be speculated that this essential partnership may not be established without face-to-face meetings. However, a process evaluation of the trial using grounded theory has shown that it is possible to establish partnerships without face-to-face meetings [32]. Although the intervention might not suit all people with COPD and CHF, those who used the platform and structured telephone support (ie, the PP group) showed a significant improvement in GSE. In addition, an increase of ≥5 on the GSE scale, which was considered a clinically meaningful improvement, was equivalent to almost 1 SD in our sample. In general, 0.5 SD is viewed as the cutoff for a clinically significant difference [33]. Previous research has shown that GSE improvement is associated with better food choices [34], improved exercise endurance in people with COPD [35], and functional fitness among older adults [36]. These findings indicate that GSE increment may support people with chronic illnesses to improve and maintain health. This study shows that PCC using a combined digital platform and structured telephone support system is one way to support people with COPD and CHF. However, this type of intervention must target those who would benefit the most from it. An important finding for HCPs is that when using self-care tools for home monitoring, our patients with CHF emphasized that they did not want to read or be reminded of CHF. This may be a way for people with CHF to cope with everyday life [8]. It also highlights the need for HCPs to listen to their patients’ concerns and individualize care accordingly.

To our knowledge, this is the first randomized trial in which the effects of PCC using a combined digital platform and structured telephone support system for people with COPD and CHF treated in primary care were evaluated. This study adds knowledge on which factors (eg, timing of the intervention and sustainability of interventional effects) are essential when designing interventions targeting self-care. In general, most self-care interventions are time intensive and require effort from the interventionist. Information on the sustainability of the effects after the end of the intervention is often not reported. Nonetheless, telehealth self-management interventions do not seem to report any adverse effects, which could suggest that such interventions are a suitable option to support people with chronic conditions [6].

### Strengths and Limitations

This study has several limitations worth noting. First, there is no information on the disease severity of CHF in the background information of the participants, as this was not consequently reported in medical records. Second, few hospitalization events indicate that another outcome measure might have been more suitable for this study population. Furthermore, the participants had a high mean GSE score at baseline, leaving little margin for improvement. The results might have been different if another outcome measure had been evaluated. Fourth, the design of the intervention might not have been ideal for all of the included participants. More participants might have been identified and benefited from the intervention if the study had used more rigorous screening criteria. Fifth, this manuscript only focuses on the effects of the intervention and might have been strengthened by adding a process evaluation; however, those data are, unfortunately, not currently available.

This study also has some strengths. First, the intervention improved GSE at the 3-month follow-up for the participants who used it. In addition, the use of telehealth made it more accessible and reduced the need for patients to travel to and from health care centers.

### Conclusions

By combining a digital platform with structured telephone support, PCC seems to be an option to increase the short-term self-efficacy of people with COPD and CHF. This study adds to the knowledge of conceptual innovations in the primary care setting to support patients with COPD and CHF. Further research is needed to explore which patient at what point in the natural history of the disease would benefit the most and tailor different digital interventions and PCC components to each patient’s unique needs.

## Appendix

Multimedia Appendix 1 CONSORT-eHEALTH checklist (V 1.6.1).

## Abbreviations

CHF: chronic heart failure
COPD: chronic obstructive pulmonary disease
GSE: general self-efficacy
HCP: health care professional
ITT: intention-to-treat
PCC: person-centered care
PP: per-protocol
